# Strategies to improve maternal vaccination acceptance

**DOI:** 10.1186/s12889-019-6655-y

**Published:** 2019-03-25

**Authors:** R. Wilson, P. Paterson, H. J. Larson

**Affiliations:** LSHTM, Keppel Street, London, WC1E 7HT UK

**Keywords:** Vaccine hesitancy, Maternal vaccination, Vaccination acceptance, Access to healthcare

## Abstract

**Background:**

In England, influenza and pertussis vaccination has been recommended for all pregnant women since 2010 and 2012 respectively. However, in some areas, vaccination uptake rates have been low. A qualitative study was conducted to gain a contextualised understanding of factors influencing vaccination acceptance during pregnancy in Hackney, a borough in north-east London, UK. This paper draws on in-depth insights gained from the above study, to provide recommendations for increasing long-term maternal vaccination acceptance.

**Methods:**

Hackney was chosen as the study site because it has one of the lowest vaccination coverage rates in pregnancy in the UK. A maximum variation sampling method was used to recruit 47 pregnant and recently pregnant women from a wide range of backgrounds, as well as ten healthcare professionals from three general practices; two community antenatal clinics; nine parent-toddler groups; and four community centres. In-depth interviews and a video-recording of a pregnant patient’s consultation, explored experiences of care within the National Health Service during pregnancy, and women’s views about maternal vaccination. In-depth interviews with healthcare professionals explored their views towards, and how they discuss and provide maternal vaccination. Study data were analysed both deductively, through drawing on insights from anthropological works that address diverse conceptualisations and practices around vaccination; and inductively, with a thematic analysis approach.

**Results:**

The findings of this study and the recommendations based on them were divided into five broad themes: access to maternal vaccination; healthcare institution rhetoric and its effect on maternal vaccination acceptance; community and family influences on maternal vaccination decisions; healthcare professionals’ views towards maternal vaccination; and the influence of patient-healthcare professional relationships on maternal vaccination acceptance.

**Conclusions:**

The strategies to improve maternal vaccination acceptance recommended in this paper would engender a more open and democratised healthcare system.

**Electronic supplementary material:**

The online version of this article (10.1186/s12889-019-6655-y) contains supplementary material, which is available to authorized users.

## Background

In England, influenza vaccination was first recommended to all pregnant women, irrespective of gestational age in November 2010 after the 2009 influenza A(H1N1) virus outbreak [1]. Additionally, in response to a pertussis outbreak in 2012, which resulted in 14 infant deaths, in October 2014 the UK Department of Health introduced the (low dose) diphtheria, tetanus, acellular pertussis and inactivated polio vaccine (dTaP/IPV) (commonly known as the pertussis vaccine). The vaccine is recommended for all pregnant women from the 16th week of pregnancy [2].

For the majority, vaccination is part of an established healthcare routine. However, despite assurances of the efficacy and safety of the maternal dTaP/IPV and influenza [3], there are many challenges to obtaining optimum vaccination rates during pregnancy. In England, the influenza vaccine uptake rate in pregnancy is 45% [4] and for the dTaP/IPV vaccine it is 74% [5].

The term *vaccine hesitancy* is used in this paper to explain one’s decision not to vaccinate, to partially vaccinate, or to delay vaccination, and is defined by The Strategic Advisory Group of Experts on Immunisation (SAGE) Working Group as,

A delay in acceptance or refusal of vaccines despite availability of vaccination services. Vaccine hesitancy is complex and context specific varying across time, place and vaccines. It includes factors such as complacency, convenience and confidence [6].

Some, like Ulrich Beck, see vaccine hesitancy as a manifestation of a broader ‘age of anxiety’ afflicting contemporary western society, and believe that we lack trust in various institutions more often today than in the past [7]. This view imagines vaccine hesitancy as new and ignores the fact that anxiety around vaccination in Britain has existed since the early 1800s, with the introduction of the smallpox vaccine [8]. Vaccination at this time attracted considerable concerns (especially surrounding libertarian arguments), and resistance [9].

The generalised rhetoric of a recent loss or breakdown of trust also does not answer many questions, such as what exactly trust is, and how it should be conceptualised. This causes problems with the ways that notions of trust, risk and resistance are leveraged in dominant policy arguments. Emphasising the negative, such as deficits of public trust, obscures people’s thoughts and actions surrounding vaccination; how people’s socio-economic positions influence vaccine acceptance and how they are treated in healthcare settings; and how vaccination makes sense within people’s everyday lives, experiences and values. For example, the worry about receiving too many vaccines could relate in some contexts to an individual focus on “overloading the immune system” [10]. However, in a more socio-political sense, it can also be argued to echo everyday experiences and concerns with unpredictable and complex government, corporate, and technical systems [11], especially today in the UK, where funding cuts to the National Health Service (NHS) are greatly affecting the quality of, and access to healthcare [12], and are a source of considerable public concern.

Additionally, discourses about loss of trust impose a normative vision of the state and the pharmaceutical industry as technocratic, trustworthy and a-political [9], so that when the public are suspicious of certain technologies, they are perceived as irrational or ignorant. Along a similar vein, research focusing on people’s engagement with vaccines has been dominated by analysis of the direct influences on their choices, in particular scientific and media information, which have led health policy to focus on information and education campaigns, which normally focus on the benefits of vaccination, and the risks of diseases [13]. Discourses about risk are beneficial to institutions promoting vaccination because they imply predictability, control and manageability, which is important given the large-scale universal aspirations of mass immunisation. However - for pregnant women in particular - this approach causes (reproductive) risk to be individualised; focusing on pregnant women’s ‘chosen’ behaviour as the primary site where reproductive risk ought to be rationally self-managed to ensure the optimum health of her foetus, without appreciating the socio-political nature of decision-making [14].

Such discourses make vaccination decision-making during pregnancy particularly difficult. Ultimately, they miss the disconnect between people’s own framings and expectations of vaccines, and those of the institutions involved in providing them. In reality, decisions regarding vaccination are not always based upon conscious deliberations of available information and calculable probabilities [15], but are made based on personal and family health histories, birth experiences (in the case of maternal vaccination), social relations. As perceived risks (such as vaccine side effects) often require expert identification and calculation (meaning that people must rely on expert advice about what risks are prevalent), people are aware that experts disagree with each other, that science and technology often generate risks, and that there are conflicting business, political and financial motives in the development and delivery of healthcare technologies. As a result, people are challenged by continued uncertainties about what information and advice to trust [16]. Vaccination decisions are thus also based on critical engagements (or disengagement) with local and national political histories, and the legacy of particular interactions between populations and institutions of the state, science, and the media [9]. Through this study, the idea that vaccine questioning or refusal is not simply a resistance to science and medical technology, but is social, political, extremely varied, complex, and context-specific is thus highlighted.

The aim of the study was to gain a contextualised understanding of access to, and attitudes towards maternal vaccination among pregnant and recently pregnant women and healthcare professionals in Hackney, London. By drawing on in-depth insights gained through analysing the socio-political context of vaccination and individuals, strategies to increase long-term maternal vaccination acceptance are suggested. The latter aspect is the main focus of this paper.

## Methods

### Study site

The London borough of Hackney was chosen as the study site as it has one of the lowest vaccination coverage rates in England [17], including for maternal vaccination. The most recent data shows that the maternal influenza vaccination coverage rate is 32% in Hackney [18] and the maternal dTaP/IPV coverage rate is 41% [19]. Historically, Hackney has also been very ethnically and socially diverse; inward migration dates back to the 18th and 19th centuries and currently there are significant ‘Other White’, Black and Turkish/Kurdish communities in Hackney [20]. The borough also has significantly more people of the Jewish and Muslim faiths than London and England in general [21].

### Recruitment

Twelve parent-toddler groups; 11 community centres and migrant support groups; and four general practitioner (GP) practices were selected as individual participant recruitment sites as - according to 2012–2014 Hackney GP practice data provided by The Blizard Institute, Barts and The London School of Medicine and Dentistry (J Robson 2015, personal communication, 13 June) - they had median maternal vaccination uptake rates and a diverse patient population. These recruitment sites were also spread across the borough, were free to attend, and attracted women from a wide range of backgrounds. It was envisaged that the above factors would make the results more generalisable). The four heads of midwifery/immunisation in Hackney were also included.

An official invitation letter explaining the study was sent by email to all potential recruitment sites. Recipients were asked to respond by email or letter if they were happy to be involved in the study. If there was no response after two weeks, RW telephoned the practice/organisation and asked to speak with the manager to explain the study and invite them to participate. RW also offered to meet them in person to discuss the study in more detail if they wished.

Maximum variation sampling was used to recruit individual participants [21]. For the recruitment of healthcare professionals and patients from GP practices and antenatal clinics included in the study, two different versions of information sheets (which requested potential participants to contact RW if they were interested in participating in the study), were sent to the practice managers. They were asked to send the relevant one to all their healthcare professionals (doctors, nurses and midwives), as well as all their currently pregnant patients, and all patients who had given birth within the past year. Women who both had and had not been vaccinated according to GP databases, were included. For the recruitment of participants from other study sites, RW sat in on sessions for parents held at parent-toddler groups and community centres and spoke to women individually, explaining the study and inviting them to participate. Posters were also put up and leaflets provided.

### Data collection

Data collection took place between December 2015 and April 2016. The methods used-in-depth interviews and a video-recording of a consultation-encouraged participants to speak widely and openly about maternal vaccination.

#### In-depth interviews

Interviews with pregnant/recently pregnant women took place at her home or a local café. A topic guide was used to elicit details of participants’ experiences of maternity care within the NHS; their views towards, and their relationships with healthcare professionals; sources of maternal vaccination information; their views towards maternal vaccination; and influences on their vaccination decisions. Interviews with healthcare professionals aimed to elicit details of their views towards maternal vaccination; how they approached the topic of maternal vaccination with their patients; whether they encouraged maternal vaccination; and what they did if a patient was hesitant, or did not want to vaccinate. Each interview was digitally recorded and transcribed in its entirety.

#### Consultation video-recording

A video recording of a consultation between a pregnant woman and her healthcare professional was conducted at the patient’s 16-week pregnancy check. Neither participant had previously been interviewed for this study. The consultation was recorded using an i-pad owned by the GP practice. Through watching the recording after the consultation had taken place, RW observed aspects of the patient-healthcare professional interaction in context; how the GP approached the subject of maternal vaccination; and the patient’s reaction to this. Although the whole consultation was recorded, only the vaccination discussion was transcribed due to its relevance to the study.

### Data management and analysis

A thematic analysis was conducted to identify, analyse, and report patterns (themes) from the data [22]. Thematic analysis was used as a ‘contextualist’ method, acknowledging the ways that individuals perceive and make meaning of their experiences, and, in turn, the ways the broader social context impinges on these meanings [23].

All interview transcripts were imported into NVivo11; a qualitative data analysis software. RW initially read the transcripts several times to become familiar with the content. They were then organised and coded into manageable text segments with the use of a coding framework and cross-checked by PP and HL. As the study began with some key questions regarding trust in vaccination and health authorities, the coding framework was formulated both deductively (through pre-established concepts guiding the research questions), and inductively (on the basis of salient and recurrent themes identified in the data).

## Results

### Recruitment

#### Study sites

Of the four GPs/practice managers contacted, three agreed to be involved in the study. The GP who declined said this was due to time constraints. Through contacting the four heads of midwifery/immunisation in Hackney, it was agreed that two community antenatal clinics could be involved in the study. Out of 12 parent-toddler groups contacted, nine be involved in the study. Four out of 11 community centres and migrant support groups contacted agreed to be involved. This amounted to 18 study sites across Hackney.

#### Interviews

Through recruiting from the above study sites, 71 pregnant and recently pregnant women showed interest in the study. However, 31 consequently did not respond to follow-up texts or declined to take part. After interviewing the remaining 40 women, saturation was reached recruitment ended. Participants were between age 18 and 41, and were from a wide variety of backgrounds. Interviews on average were an hour long.

Ten healthcare professionals responded to the invitation letters sent through the three GP practices and two antenatal clinics and were included in the study. All healthcare professionals were female, between ages of 23 and 62, and had been in their current role for between six months and 35 years. Six participants were GPs, two were midwives and two were practice nurses, and were from a variety of ethnic backgrounds. Interviews on average were 20 min long.

Demographic details of all participants, as well as pregnant/recently pregnant participants’ vaccination status is provided in Additional file 1.

#### Video-recording

One woman’s 16-week (pregnancy) consultation was video-recorded. This participant was in her mid-thirties and White British. The consultation lasted 21 min and the vaccination discussion lasted about four minutes.

Fifty-seven participants were recruited in total.

### Major findings

#### Access

Middle-class women who were citizens of the UK tended to believe that they had all the vaccination information that they needed; indeed some even felt overwhelmed by such information from leaflets and online research. However, some women who were more marginalised, especially those whose first language was not English (such as Japanese mother Tami; Turkish mother Sabah; and Orthodox Jewish mothers Talia and Meira), found it difficult to understand verbal vaccination information, especially if their healthcare professional had a strong accent or used medical ‘jargon’. Some women, like Tami, were embarrassed about asking for clarification. Sabah even avoided antenatal clinics due to not speaking fluent English. Additionally, Talia had a baby to look after, and so could not attend vaccination appointments.

#### Healthcare rhetoric

Five Black British Caribbean participants were hesitant to vaccinate. These women had fears that the vaccines were “something that the government are putting in people” (Renee, Black British Caribbean midwife), and worried that vaccines can affect various populations differently (Tessa, Black British Caribbean mother). For four of these participants, as young, unmarried, unemployed Black Caribbean mothers, it is possible that the intersection of their socio-economic position, ethnicity, and gender had consequences regarding their treatment by government services, which can see women with such identities as irresponsible and in need of management,

I was told, if you don’t... *make* [emphasis added] the child get all their injections that… they can… bring up, like, my background… see if I had a social worker, and social worker can get onto my case because... it’s like I’m not protecting the child… that’s what I was told by one of my midwives… she’s the one that closed my case, after I told her, “yes, [my daughter] will get all her, um, injections”… These injections are new to me, so for me to just say, yeah, I’m going to give it to [my daughter], it’s something that I was kind of being forced to do, without... thinking about it… and the same GP… she told me that you need to… and I-I felt intimidated… under pressure because she was telling me that… these are the things that can happen if you don’t get the child [vaccinated] (Jane, Black British Caribbean mother).

The threat that the state would be involved in the form of Jane having a social worker “on her case” if she did not vaccinate her child, demonstrates one of the ways in which women are punished for failing to conform to ideologies of ‘being a good mother’. This is the case particularly for women marginalised or discriminated against due to their ethnicity and socio-economic position. Rebecca, a middle-aged, married, self-employed White British mother who refused vaccination for both her children, faced no such threats.

There also existed a strong desire for support in making healthcare decisions among marginalised women,

I went in to be checked they said they’re not sure if it was my waters [broke]… You expect when you’re going into someone’s care for them to say, “Right well this is what’s happening”. Not, “What do you want to happen?”… eventually a midwife, because I broke down in tears and I was like, “No-one knows what they’re doing in here” and then um she was like… “what if I make the decision for you?” and I said, “Okay” and she said, “Right you’re being induced”. I said, “Alright”… She was lovely. I remember her (Ava, White British, unemployed mother).

Some women in more precarious positions even wanted to hand over certain decisions-such as about vaccination-to healthcare professionals. For example Haadiya, a young unemployed Nigerian mother, who had recently moved to the UK said, “my first midwife… said just use NHS [website] otherwise its confusing, and I do. It’s… just all so contradictory. Someone has got to make a decision for you”.

#### Community and family influences on vaccination decisions

When asked who made decisions about their health, all 40 women interviewed immediately and usually proudly, responded “me”. However, later in the interview, when asked specifically if there was anyone or anywhere they would typically go to for advice regarding their health or vaccination, all participants mentioned friends or family members, (usually female family members such as sisters who already had children, mother-in-laws, mothers and aunts), as well as, or rather than their GP. For example, Jane said that now that she had heard about the maternal influenza vaccine from Jamaican friends and family, she would accept it if she was pregnant again.

Despite declining maternal vaccination, Shiloh, as well as some other participants were angry that their infants were too old to receive the newly introduced meningitis B vaccine [24]. The fact that her nephew had received the meningitis B vaccine, and the vaccine had featured in the news a lot around this time, could have normalised it and reassured Shiloh of its perceived safety and necessity. In contrast, the maternal dTaP/IPV and influenza vaccines had not been discussed in detail with Shiloh.

Ten participants had family members who were healthcare professionals, whose vaccine advice was trusted more than advice given by non-related healthcare professionals. Such advice often carried warnings not to vaccinate,

My sister [who is a midwife]… [said] testing vaccines in pregnancy is really limited… so that really put me off… when you’re told by doctors and nurses that you need to have that it’s quite difficult to… argue unless you… have somebody, like I have my sister who is a midwife… I would’ve trusted my sister more for honest advice because… [healthcare professionals would] be promoting all the… injections… and I’ve read lots of stories of children being really unwell after having them… a doctor, a nurse wouldn’t tell you that (Anna, White British mother).

Additionally, the parents of women who are currently offered maternal vaccination are of a generation that pre-dates it, and so participants often reported that their mothers had told them that pertussis and influenza are common, harmless diseases.

Participants with male partners tended not to seek advice from them as much as from their female friends and family members, and so in many instances, they were not engaged in vaccination decisions. Some women actively excluded male partners from the decision-making process, “My partner doesn’t factor in... When it’s inside me, it’s my baby” (Rita, White/Jewish British mother). However, the fact that male partners were not involved in vaccination decisions may not have always been the woman’s choice, and may have sometimes placed an extra burden of responsibility on her. Rebecca recalled, “I don’t really think I spoke to my husband about [vaccination] [Laughs]. Because, he’s really busy” (Rebecca, White British mother).

#### Healthcare professionals’ views towards maternal vaccination

Healthcare professionals were generally pro-vaccine, however, some held concerns or misconceptions about the vaccines. For example, both a GP and a midwife were concerned that the influenza vaccine could cause influenza and worsen symptoms. They were also not convinced of the vaccine’s efficacy. The same midwife was concerned about the fact that the dTaP/IPV vaccine contained antigens other than against pertussis. Another GP was concerned about the tetanus antigen of the dTaP/IPV vaccine; worrying about the effects of receiving too many tetanus vaccines during a lifetime. Additionally, a mother who was a nurse believed that the influenza vaccine was only provided so that healthcare professionals did not “go off sick”, rather than to protect pregnant women; thus believing that the government had ulterior motives for promoting vaccination. Most healthcare professionals also believed that the dTaP/IPV vaccine was more important than the influenza vaccine. One midwife was personally against vaccination in general, and lacked knowledge about maternal vaccines and the diseases they prevent.

Most healthcare professionals asserted that they recommended the maternal influenza and DTaP/IPV vaccines. However, later in the interview when they were asked about their recommendation specifically, it often became apparent that they did not actively recommend the vaccines, but merely mentioned them. Additionally, only one GP interviewed mentioned that vaccination prompts (through IT systems) were available at her practice. These observations are important considering that most women said that they would have vaccinated if they had been offered by and discussed with their healthcare professional.

While the two midwives said that they administered vaccines, two GPs and a practice manager said that many midwives are not trained to administer vaccination. This issue caused frustration among GPs and pregnant women alike, as it meant women having to book extra appointments with GPs or nurses to receive the vaccines, which was inconvenient or forgotten. Both GPs and women also mentioned the issue of miscommunication around maternal vaccination; there was a concern that this led to healthcare professionals believing that another healthcare professional had recommended vaccination, but in reality, nobody had.

#### Patient-healthcare professional relationships

A number of pregnant/recently pregnant participants reported grievances that related to pressures and time constraints facing healthcare professionals, including not receiving appointment letters, long waiting-times, feeling rushed and being in a chaotic care environment.

Most healthcare professionals stated that they reassured vaccine hesitant women of the safety of the vaccines, and offered to discuss vaccination with them further if they had any concerns. This was evident in the consultation that was video-recorded: Dr. Shaw employed a participatory approach to the vaccination discussion, which invited her patient into the conversation, while also asking her about wider aspects of her wellbeing. However, according to the women interviewed, this rarely happened, especially if they did not initiate the conversation themselves. Instead, women stated that they were often handed leaflets, or advised to conduct online research. Almost all women interviewed stated that they would have liked to have a more in-depth, verbal conversation with their healthcare professional about their concerns, rather than just being given information about vaccination. Lucy (age 27) expressed the want for healthcare professionals to take time to discuss her concerns and ask her what she was comfortable with “so that they can actually understand you as a person”. However, instead of feeling reassured, many women, especially if they were young, single and/or unemployed, reported feeling judged by them, or that their concerns were dismissed. This was not helped by the fact that most women saw many different midwives throughout their pregnancy, meaning it was difficult to build relationships with them and trust their vaccination advice.

For detailed results of this study, please refer to Wilson, 2018 [25].

## Discussion

### Access

Programmes should be developed that aim to change factors that engender inequalities in access to healthcare, rather than default to strategies of individual risk management, which blame individuals for inequalities in access to healthcare [26] and can lead them to disengage with the healthcare system.

While language or cultural ‘barriers’ are often blamed for under-vaccination, it is more productive to examine communication issues that could lead to a lack of knowledge, misconceptions or distrust in healthcare professionals or vaccination that arise within the healthcare system. Even English speakers report communication challenges when trying to navigate the NHS. The fault for low vaccination uptake rates among certain groups is thus more likely to lie within the healthcare system and its failure to engage certain population groups. For example, it was previously assumed that the Charedi community in North London had systemic religious or cultural objections to vaccination, but it has recently been shown that this is not the case [27]. Instead, in my study, the two Orthodox Jewish participants faced structural barriers to accessing vaccination. One was not able to attend the vaccine appointments due to having another baby at home to take care of, and the other did not understand what maternal vaccination was for due to it being explained in English without a translator present.

To address issues of miscommunication, translation services should be enhanced and maternal vaccination leaflets should be translated into a variety of languages. Additionally, technical terms used in medical settings and in vaccination promotion materials should be translated into lay language that is culturally appropriate.

Uniformity in access to vaccination could be partly achieved through standardisation in the organisation of services. For example, IT systems could provide prompts to identify when pregnant women are eligible for the vaccines so that reminder letters can be sent. GPs could also telephone women to discuss vaccination directly with them. Additionally, a maternal vaccine helpline could be established, so that women can easily find accurate information and reassurance about vaccination.

It is important that women are clear about what exactly the vaccines offered immunise against, so that they do not feel that information is being hidden from them. Maternal vaccination should also be more widely publicised.

As was recommended by many participants, the maternal dTaP/IPV and (in influenza season), the influenza vaccine, should be recommended at a particular point during a woman’s pregnancy, and administered together at the same appointment in which they are recommended, in order to save healthcare professionals time, and for the convenience of the pregnant woman. However, it is important that in the influenza season, women do not delay receiving the influenza vaccine until 16 weeks of pregnancy (the time from which the dTaP/IPV vaccine should be provided) [28]. Additionally, waiting times for appointments should be reduced in order to make it easier for, and to encourage pregnant women to attend appointments.

Ultimately, GP practices and antenatal clinics should have specific but aspirational targets for maternal vaccination. This could involve having a ‘vaccine champion’-a member of staff who oversees and creates enthusiasm for vaccination campaigns, and encourages improved communication about vaccination between healthcare professionals. This could be somebody like Midwife Williams,

I encourage [vaccination] because I do believe in it, and… I’ve got some leaflets and… I went to [management], I said, “Look, we do need more leaflets.” I was the one that requested that when they ran out".

Through information systems that produce data on access to healthcare, as well as experiences of care among various population groups, services could promote and monitor equity in access to and quality of healthcare [29].

### Healthcare rhetoric

Those deciding not to vaccinate are often seen as ignorant or deviant, as was the case when Jane was threatened with social services when she considered not vaccinating her daughter. The blaming of women who already face discrimination due to their socio-economic position, does little to encourage them to vaccinate. In ignoring individual women’s histories and experiences, not only are their perspectives misunderstood and thus they become alienated, but flawed and ineffective policies are introduced, thus perpetuating the problem. For example, the current focus on presenting the public with copious amounts of information based on ‘scientific facts’ in order to increase vaccination acceptance, assumes public ignorance and a lack of rational thinking, and thus contributes to the stereotypes often applied to vaccine hesitant women by healthcare institutions and professionals. Additionally, partial accounts of success stories about controlling disease through vaccination conceals complicated histories, masks problematic collaborations with pharmaceutical companies as well as failures in science, and ignores external socio-political factors that affect healthcare [30]. Instead of being presented with abstract statistics with which they are expected to make probability calculations in order to make decisions, most pregnant women want verbal discussions, which include reassurance and empathy. As decisions occur socially and often do not account for clear certitudes and scientific explanations [31], a narrative approach from some*one*, which generates emotions, can be more effective in encouraging vaccination acceptance than presenting ‘facts’ verbally or through some*thing*, such as leaflets. Vaccination promotion materials should therefore be used as a supplement to more in-depth and personalised vaccination discussions.

Along a similar vein, the use of alternative therapies should not be dismissed in negative terms, as they are often used alongside vaccination, and the therapists involved can be an important source of support [13]. This means that if women engage with alternative therapists or mobilisation networks around vaccination, they should be discussed, and their role understood.

Additionally, at a time where individualist notions of healthcare are popular, and where patients believe that they are the experts of their own health, information should be presented in a way that affirms self-worth or core values. It should acknowledge the value of women’s expertise; their dedication to their and their foetus’ health; their commitment to active health seeking; and what they already do to protect their and their foetus or infant’s health [9].

The language of herd immunity should be reconsidered. When used with respect to public health, the term ‘herd’ conjures images of herds of sheep or cattle blindly following orders. This blind acceptance of authority is one of the fears that some vaccine resistance is based on. Such language is also too abstract and insensitive to relate to particular women and their infants, and dismisses women’s individual perspectives. A more appropriate term to explain that optimum vaccination rates are necessary to protect the whole population, may be ‘community immunity’, as it conveys the idea of caring for others within the community.

The top-down approach to vaccination policy currently pursued by PHE jars with the currently popular individualised notion of healthcare and the rhetoric of patient choice. Greater public involvement in decisions around vaccination policy and programmes would encourage a move away from expectations of compliance, to concordance. This would allow space for individuals’ own definitions of health-which are relative, dynamic and strongly linked to personal experience-without removing them from the broader social context [32]. Such an approach could take the form of PHE creating working groups where healthcare professionals, concerned publics, and social science academics are invited into the design process of vaccination campaigns. A similar approach, with a focus on “patients as partners” was conceptualised and brought together as a programme by the King’s Fund, and has been implemented by a number of NHS Trusts since September 2016 [33].

Additionally, organisations that do not support their healthcare professionals are unlikely to prioritise respectful care for women [34]. It must be taken into account that health systems constraints such as underfunding (as currently faced by the NHS), can frustrate the workforce and undermine healthcare professionals’ performance and professionalism, as well as their sense of ‘good will’. Given the well-established link between staff wellbeing and the quality of patient care, maintaining a healthy workforce as the NHS goes through a period of intense pressure is therefore particularly important.

Overall, vaccine hesitant women, as individuals who are normally open to discussing vaccination, should be the target of efforts to increase maternal vaccination acceptance, rather than vocal deniers. This is because when individuals have strong beliefs about something, they often hold onto these beliefs, even when the evidence for them is refuted [35].

### Involving community and family members in vaccination decisions

Friends and family members had an overwhelming influence on participants’ vaccination decisions. Often participants sought advice from these contacts not because they were looking for factual information, but because they wanted personally focused discussions and advice and to feel comfortable and cared for [36]. It is therefore important that maternal vaccination promotion material reaches a wider public than pregnant women, especially as participants often mentioned family members who had negative views towards the vaccines. Family members and friends could thus be encouraged to be involved in vaccine discussions and decisions as much as possible, for example if they attend consultations with the pregnant patient, if of course, this is what the patient wants. Particular efforts should be made to include male partners in such discussions.

Partners and other influential family members should also be better represented in vaccination promotion material. Currently, the NHS maternal vaccination promotion leaflets and posters contain a photo of a pregnant woman (on her own) on the front page, with the leaflet for influenza vaccination entitled, “Flu, *your* pregnancy and *you”* [emphases added] [37]. The lack of representation of other social contacts, and the emphasis of the effects of influenza only on the individual pregnant woman, excludes others invested in her and her infant’s health, and who may influence decisions around such matters.

Including these contacts in vaccination decisions could dispel traditionally held misconceptions about the vaccines, possibly making it less likely that they will try to persuade the pregnant woman against vaccination, and would enable them to feel more included in her healthcare. This approach may even lead to friends and family members encouraging vaccination; something which was experienced by one participant (Isleen), which led her to realise that it would be a good idea to receive the pertussis vaccine during her pregnancy.

Additionally, as the parents of women who are currently offered maternal vaccination are of a generation that pre-dates it, it is especially important that they are aware of the maternal vaccine recommendations and the importance of the vaccines.

### Healthcare professional’s views towards maternal vaccination

Healthcare professionals should receive training so that they understand the importance of vaccination; have the chance to discuss any concerns they may have; and ensure that they can manage the high expectations of the system and the demands and questions of patients.

As midwives usually have close relationships with pregnant women, they should be more involved with vaccine programmes and promotion. All midwives should receive appropriate vaccination training so that women can receive the vaccines when they are recommended, rather than having the inconvenience booking extra appointments to receive them. This is essential because under the emerging health service framework in England, with the growing shift away from hospitals and GPs to community-based services such as separate midwife-ran maternity services, midwives will have greater roles, responsibilities and influence [38]. Along these lines, discussions should be had with healthcare professionals about trends towards growing privatisation within the NHS, and its implications for vaccination.

### Patient-healthcare professional relationships

While healthcare professionals should be knowledgeable and provide advice based on scientific evidence, it is also important for them to build open, trusting relationships with pregnant women [39]. These relationships could be built through training healthcare professionals to be more empathetic and to encourage dialogue when recommending vaccination. This is extremely important as most women said that they would have accepted vaccination if it had been offered by and discussed with their healthcare professional. Tailoring vaccination discussions in this way does not need to be so individualised that it is inefficient to the healthcare service. In fact, a method of “mass customisation” can be employed where individuals can be grouped according to their expressed preferences, and methods of personalisation can be systematised [40].

The way in which the topic of vaccination is initiated can significantly affect patient’s vaccination decisions [41]. An example of a positive approach to a vaccination discussion was that used by the GP whose consultation was video-recorded, who employed a participatory approach. In contrast, GPs also often use a presumptive format, involving asserting a position regarding vaccination (for example, in my study, Ava (age 26), said that her healthcare professional stated, “Right, are we going to get the vaccinations?”). This format can constrain women’s participation and thus only be beneficial in certain situations. For example, if it is used by healthcare professionals who have knowledge and an established relationship with their patient to determine that a non-participatory initiation of the topic of vaccines is appropriate [41]. This approach could also be employed to oppose the currently popular individualised model of healthcare, which encourages leaving patients to make their own healthcare choices, and was important to all healthcare professionals interviewed. However, due to public health institution’s need for high vaccination rates, and varying social positions of individuals, completely hassle-free, free choices are not an option for all women. Such an approach can also be perceived as neglectful. Some women, like Ava preferred help in making such decisions.

An example of a relational, participatory approach to structuring the vaccination conversation with vaccine hesitant women in consultations is as follows,

1. Healthcare professionals should introduce themselves to their patient and explain what they can expect from the consultation
2. Explain what the vaccines are and why they are important
3. Check the patient’s decision making role preference (i.e. involving her to the extent that she desires)
4. Explore expectations and any fears surrounding vaccination
5. Provide personalised information and reassurance based on the patient’s concerns (acknowledge or be honest if an answer to a patient’s question is not known)
6. Discuss potential options for moving forward (such as having time to think about the decision, coming back to discuss it further if necessary, and not pressuring the patient to vaccinate)
7. Check the patient’s understanding of information and her expectations of options
8. Support the patient to make a decision.

(Adapted from Elwyn and Charles, 2001 [42]).

A relational approach would also require more midwife continuity, so that the same one or two healthcare professionals spend more time with women over the course of their pregnancy in order to build trusting relationships and enable any concerns to be discussed fully.

Most of the suggestions mentioned require additional funding to be directed at providing longer consultations and the training of healthcare professionals towards a more relational approach to care. Ultimately, this could lead to consistent, equitable, and high levels of care, and thus increased levels of vaccination acceptance.

### Strengths and limitations

The methods used allowed for an in-depth analysis of the socio-political specificities in the lived reality of maternal vaccination, in order to provide anthropologically informed recommendations about how to increase acceptance of, and access to vaccination.

This study is the first to provide an analysis of attitudes towards maternal vaccination in Hackney, and is also (to our knowledge), the first anthropological study analysing views towards maternal vaccination. The results could thus be used to develop appropriate and tailored policies for increasing vaccine acceptance within, and outside of the UK, especially as an increasing number of countries (especially in the developing world) are introducing maternal vaccination.

A literature review showed that very few studies take men’s views towards maternal vaccination into account [43]. RW therefore aimed to include such perspectives, through asking participants’ male partners if they would also be happy to be interviewed when visiting participant’s homes to conduct the interviews. However, usually only the participant was at home and so this was not possible.

A small number of healthcare professionals were interviewed and their interviews were relatively short due to difficulties in recruiting such a time constrained cohort. However, enough information was gathered in order to effectively inform the findings of the study. Additionally, all healthcare professionals interviewed happened to be women. This may be because healthcare professionals who treat pregnant women are more likely to be women [44].

The Hawthorne effect [45] may have occurred during the consultation video-recording. However, various studies have shown that neither consulting nor patients’ behaviour is affected by their awareness of the recording [46, 47]. Additionally, the NHS ethics committee believed that recording the consultation through an i-pad would be less intrusive than if observing the consultation in person. However, when they were invited to participate in the study by having their consultation video-recorded, women often said that they were worried that the recording could end up online. Therefore, only one consented to having her consultation recorded. This method was thus used as a supplementary illustrative aspect of the study.

## Conclusions

An equitable healthcare service would meet healthcare needs across the population, and ensure uniformity in access, use, and quality at the point of delivery, through flexibility as well as standardisation in the organisation of services. This would mean that women experience predictability and consistency in the care that is provided. The relational approach to healthcare, which requires support and close relationships between healthcare professionals and patients, would engender an understanding of women’s experiences and perceptions in context, and enable them to be more involved in healthcare decisions. It would help to address the assumptions and normative frameworks underlying healthcare provision, which, whether due to resistance to such frameworks, alienation, or discrimination within the healthcare system, can exclude women from vaccination. Following this, a move should be made away from moralising individual behaviour and encouraging individual women to change, to addressing the deeper, structural conditions that affect women’s and their broader collectives’, choices and actions.

Additionally, ethnographic engagement in various healthcare settings, allowing the space for dialogue with pregnant women and the telling of their stories and experiences would allow for wider conceptualisations of healthcare and vaccination across various communities. This would mean that vaccine hesitancy can be more deeply understood by healthcare institutions, professionals, and academics.

These approaches would engender a more open and democratised healthcare system. It would mean that care is shared rather than individualised, and so would reduce the focus of responsibility placed on pregnant women, who’s bodies undergo more stringent (self)-management, analysis and scrutiny than any other bodies.

## Additional file

Additional file 1: Participant demographics and vaccination status of pregnant/recently pregnant women. (DOCX 24 kb)

## Abbreviations

dTaP/IPV vaccine: Diphtheria, tetanus, acellular pertussis and inactivated polio vaccine
GP: General practitioner
NHS: National Health Service
PHE: Public Health England
UK: United Kingdom
WHO: World Health Organisation
