# Supplementary material for: Strategies to improve maternal vaccination acceptance
Source: BMC Public Health. 2019 Mar 25;19:342. doi: 10.1186/s12889-019-6655-y (PMC6434850; doi:10.1186/s12889-019-6655-y)
Supplement: Supplementary file 1 — Participant demographics and vaccination status of pregnant/recently pregnant women. (DOCX 24 kb) [file 12889_2019_6655_MOESM1_ESM.docx]

**Appendix 1: Participant demographics and vaccination status of pregnant/recently pregnant women**

Pregnant women

| **Participant^*^** | **Age** | **Ethnicity** | **Religion** | **Education** | **Employment** | **Marital status** | **Primiparous**  **/multiparous** | **DTaP/IPV** | **Influenza** | |  |
| --- | --- | --- | --- | --- | --- | --- | --- | --- | --- | --- | --- |
| Anna | 28-30 | White British | None | Bachelor’s | Unemployed | Married | Multiparous | N | | N |  |
| Margaret | 38-48 | White British | None | Bachelor’s | Employed | Co-habiting | Primiparous | Y | | Y |  |
| Tami | 28-38 | Japanese | Buddhist | Master’s | Unemployed | Married | Primiparous | Y | | Y |  |
| Hayley | 28-30 | White British | None | Bachelor’s | Employed | Married | Primiparous | N | | N |  |
| Maddelin | 28-38 | German | None | Bachelor’s | Employed | Co-habiting | Primiparous | N | | N/a |  |
| Magda | 28-38 | White British | None | Bachelor’s | Employed | Co-habiting | Primiparous | Y | | N/a |  |
| Lucy | 18-28 | Black British | Catholic | GCSE | Employed | Single | Primiparous | Y | | N |  |
| Anetta | 28-38 | Nigerian | Christian | Bachelor’s | Unemployed | Married | Primiparous | N | | N |  |
| Bathild | 28-38 | German | None | Bachelor’s | Unemployed | Married | Primiparous | Y | | Y |  |
| Celia | 28-38 | White British | Catholic | Master’s | Employed | Married | Primiparous | Y | | Y |  |
| Ava | 18-28 | White British | None | GCSE | Unemployed | Single | Multiparous | Y | | Y |  |
| Shiloh | 18-28 | Black British Caribbean | Christian | NVQ | Unemployed | Single | Multiparous | N | | N |  |
| Caera | 18-28 | White/black Caribbean | None | GCSE | Unemployed | Single | Primiparous | Y | | N/a |  |
| Sarah | 18-28 | White British | None | GCSE | Employed | Single | Primiparous | Y | | N/a |  |
| Gabriela | 18-28 | White British | None | High school | Unemployed | Single | Primiparous | Y | | Y |  |
| Isleen | 28-38 | Australian | None | Master’s | Employed | Married | Multiparous | Y | | Y |  |
| Rebecca | 28-38 | White British | Christian | Bachelor’s | Employed | Married | Multiparous | N | | N |  |
| Ruth | 28-38 | White British | None | Master’s | Employed | Co-habiting | Multiparous | Y | | N/a |  |
| Cai | 28-38 | Chinese | Christian | Master’s | Unemployed | Married | Primiparous | Y | | Y |  |
| Haadiya | 18-28 | Nigerian | Muslim | GCSE | Unemployed | Married | Primiparous | Y | | Maybe |  |
| Tessa | 18-28 | Black British Caribbean | Christian | High school | Employed | Cohabiting | Primiparous | Y | | N |  |
| Carla | 28-38 | White British | Catholic | Master’s | Employed | Married | Primiparous | Y | | Y |  |
| Samantha | 28-38 | Black British Caribbean | Christian | Bachelor’s | Employed | Single | Multiparous | N | | N |  |
| Jane | 18-28 | Black British Caribbean | Christian | High school | Unemployed | Single | Multiparous | N | | N |  |
| Kate | 28-38 | South African | None | Master’s | Employed | Married | Primiparous | N | | N |  |
| Haleefa | 18-28 | Somalian | Muslim | Master’s | Unemployed | Married | Primiparous | Y | | N |  |
| Talia | 18-28 | Orthodox Jewish | Orthodox Jewish | High school | Unemployed | Married | Multiparous | N | | N |  |
| Meira | 18-28 | Orthodox Jewish | Orthodox Jewish | High school | Employed | Married | Multiparous | N | | N |  |
| Rafeal | 28-38 | White British (ethically Jewish) | None | Master’s | Employed | Co-habiting | Primiparous | Y | | Y |  |
| Beth | 18-28 | White British (Irish) | Catholic | NVQ | Unemployed | Single | Primiparous | Y | | Y |  |
| Mahsa | 28-38 | Pakistani British | Muslim | Master’s | Unemployed | Married | Multiparous | Y | | N |  |
| Julia | 28-38 | Chinese/English | None | Master’s | Unemployed | Single | Primiparous | N | | N |  |
| Isobel | 38-48 | White British | None | Master’s |  |  |  | Y | | Y |  |
| Marigold | 28-38 | British/Brazilian | Christian | Master’s | Employed | Married | Multiparous | Maybe | | Y |  |
| Sabah | 28-38 | Turkish | Muslim | High school | Unemployed | Married | Multiparous (4 children) | N | | N |  |
| Ezgi | 28-38 | Turkish | Muslim | High school | Unemployed | Married | Multiparous | N | | Y |  |
| Idda | 18-28 | Norwegian | None | Master’s | Student | Co-habiting | Primiparous | N | | N |  |
| Cadenza | 28-38 | Italian | Christian | High school | Employed | Co-habiting | Multiparous | N | | N |  |
| Zoe | 28-38 | Black British | Christian | Master’s | Unemployed | Single | Multiparous | Y | | N |  |
| Aldona | 28-38 | Lithuanian | None | NVQ | Unemployed | Married | Multiparous | N | | N |  |

Healthcare professionals

| **Participant*** | **Age** | **Ethnicity** | **Religion** | **Recommend vacc.** | **Encourage vacc.** |
| --- | --- | --- | --- | --- | --- |
| Dr. Lawson | 48-58 | White British | None | Y | Y |
| Dr. Marsh | 18-28 | White British | Christian | Y | Y |
| Dr. Cooke | 28-38 | White British | Christian | Y | Y |
| Midwife Williams | 48-58 | Black British | None | Y | Y |
| Dr. Henderson | 28-38 | White British | None | Y | Y |
| Dr. Khatri | 58+ | British Indian | Hindu | Y | Y |
| Nurse Anand | 38-48 | British Indian | Christian | Y | Y |
| Nurse Thompson | 38-48 | Black Caribbean | Christian | Y | Y |
| Midwife Renee | 38-48 | Black/white Caribbean | None | Y | N |
| Dr. Clark | 28-38 | White British | Catholic | Y | Y |

*All participant names used in this paper and in the tables above are pseudonyms. All participants provided both written and verbal informed consent that the information provided in the tables above can be published.
